# Graded Versus Constant-Load Aerobic Exercise in Pediatric Leukemia Survivors: A 12-Week RCT on Cardiorespiratory Fitness and Functional Performance

**DOI:** 10.3390/healthcare14050608

**Published:** 2026-02-27

**Authors:** Ragab K. Elnaggar, Ahmad M. Osailan, Ahmed S. Ahmed, Hesham A. Alfeheid, Mohamed S. Abdrabo, Heba M. Y. El-Basatiny, Gaber S. Soliman, Amira E. El-Bagalaty

**Affiliations:** 1 Department of Health and Rehabilitation Sciences, College of Applied Medical Sciences, Prince Sattam Bin Abdulaziz University, Al-Kharj 11942, Saudi Arabia; 2 Department of Basic Sciences, Faculty of Physical Therapy, Cairo University, Giza 11432, Egypt; 3 Department of Medical Rehabilitation Sciences, College of Applied Medical Sciences, Najran University, Najran 66462, Saudi Arabia; 4 Department of Physical Therapy for Pediatrics, Faculty of Physical Therapy, Cairo University, Giza 12611, Egypt; 5 Department of Physical Therapy and Health Rehabilitation, College of Applied Medical Sciences in Al-Qurayyat, Jouf University, Al Qurayyat 77454, Saudi Arabia; 6 Department of Physical Therapy for Cardiovascular/Respiratory Disorder and Geriatrics, Faculty of Physical Therapy, Cairo University, Giza 11432, Egypt

**Keywords:** acute lymphoblastic leukemia, aerobic exercise, cardiopulmonary fitness, functional performance, rehabilitation, survivorship

## Abstract

**Background:** Cardiorespiratory fitness is frequently impaired in survivors of pediatric acute lymphoblastic leukemia (ALL), limiting their functional performance. While aerobic exercise is recommended, evidence is needed to guide the prescription of specific training protocols in this population. **Objective:** This study sought to compare the efficacy of constant-load (CL-AEx) and graded aerobic exercise (G-AEx) protocols on cardiorespiratory fitness and functional capability in pediatric survivors of ALL. **Methods:** Seventy-two pediatric ALL survivors were allocated to CL-AEx, G-AEx, or a control group. Cardiopulmonary fitness [peak oxygen consumption (peak VO_2_), peak minute ventilation (VE), ventilatory equivalent for oxygen (VE/VO_2_), respiratory exchange ratio (RER), peak oxygen pulse (peak O_2_P), maximum heart rate (max HR), and one-minute heart rate recovery (HHR_1_)] and functional performance [six-minute walk test (6MWT), 4x10-m shuttle run test (4x10-mSRT), and timed up down stairs (TUDS)] were assessed at pre- and post-intervention. **Results:** The G-AEx group exhibited significantly enhanced cardiorespiratory and functional outcomes compared to both the CL-AEx and control groups (all *p* < 0.05). The G-AEx group demonstrated more pronounced improvements, showing significant increases in peak VO_2_, VE, VE/VO_2_, peak O_2_P, and HHR1, alongside a more efficient RER. Functionally, the G-AEx intervention led to superior improvements in 6MWT distance, and significantly faster completion times in the 4x10-mSRT and TUDS, highlighting multi-domain functional gain. **Conclusions:** In pediatric survivors of ALL, G-AEx demonstrated superior improvements in cardiorespiratory fitness and functional performance compared to CL-AEx over 12 weeks. These findings suggest that G-AEx is an effective modality for addressing acute physical deconditioning in this population. Incorporating G-AEx into clinical rehabilitation may enhance immediate physiological and functional recovery during the survivorship phase.

## 1. Introduction

Acute lymphoblastic Leukemia (ALL) is the leading childhood malignancy, accounting for nearly 80% of pediatric leukemias and 25% of all childhood cancer cases globally [1]. Advances in treatment over the last 10 years have significantly enhanced outcomes, with a five-year survival rate of 90.4% [2]. Nevertheless, these improvements in survival are accompanied by notable challenges and long-term implications. Survivors are at risk of a variety of complications spanning short-, intermediate-, and long-term periods, stemming from the disease itself or chemotherapy exposure. These may involve cardiorespiratory challenges, neurological deterioration, cognitive decline, musculoskeletal issues, disruptions in growth and pubertal progression, and overall physical deconditioning [3,4,5,6]. This has led to a heightened focus on approaches that prioritize health promotion and quality in survivors.

Research indicates that cardiorespiratory function and physical fitness are adversely affected during and after ALL treatment [4,7,8,9]. These fitness domains serve as essential health indicators, signifying the functional capabilities of numerous bodily systems engaged in routine physical activities. Therapeutic interventions for pediatric cancers, such as chemotherapy or radiotherapy, can inflict acute and chronic harm to the heart, lungs, and skeletal musculature, which are vital for maintaining optimal fitness levels [7,9,10,11]. Moreover, the sedentary behavior often associated with the treatment of pediatric cancers leads to cardiorespiratory and overall physical deconditioning [7,9]. These effects limit the ability to engage in life roles and recreational pursuits that are contingent upon good fitness levels, accentuating the need for holistic rehabilitation strategies that address all facets of health and functionality.

Current rehabilitation paradigms for pediatric survivors of ALL emphasize functional optimization and community reintegration through targeted interventions. Therapeutic exercise regimens and structured physical activity programs have empirically been supported for implementation throughout the complete treatment trajectory, spanning intensive chemotherapy to maintenance therapy and subsequent survivorship care [12,13]. Emerging studies suggest that exercise-based interventions generally contribute to improvements in cardiorespiratory fitness, muscle strength, fatigue perception, flexibility, and functional capacity [12]. Among various exercise modalities, aerobic training has been particularly beneficial for ALL patients and long-term survivors. Such an exercise approach, whether implemented independently or integrated with other modalities, has been empirically validated as a highly effective intervention with wide-spectrum health benefits [8,12,14,15,16]. Yet, the question of the optimal training strategy—particularly in terms of intensity, duration, volume, and progression rate—remains unresolved. Among the frequently implemented strategies are constant-load (CL-AEx) and graded (G-AEx) aerobic exercise. CL-AEx involves maintaining a fixed intensity throughout the program [17], which can be effective for inducing initial adaptations. However, without adjustments, the relative training stimulus diminishes as an individual’s fitness improves, potentially leading to a plateau in physiological gains. In contrast, G-AEx is an inherently progressive strategy structured around controlled, incremental increases in training parameters such as intensity or duration [8,18,19,20]. This approach is built upon the fundamental principle of progressive overload, which posits that for adaptations to continue, the physiological stress placed on the body must gradually increase over time. For pediatric ALL survivors, who often present with significant deconditioning and a heterogeneous range of physical capabilities [3,4,7], a graded protocol may offer distinct advantages. The initial lower-intensity phases can enhance safety, build self-efficacy, and improve training tolerance, while the subsequent, structured progressions ensure that the training stimulus remains challenging enough to drive further improvements in cardiorespiratory fitness [8]. Furthermore, the varied and progressively challenging nature of G-AEx may also enhance participant engagement and motivation, potentially leading to better long-term adherence compared to more monotonous, steady-state protocols [21].

While prior investigations have confirmed the general effectiveness of aerobic exercise for pediatric ALL survivors [8,12,14,15,16,22], evidence directly comparing the efficacy of graded versus constant-load protocols in this specific population is lacking. This knowledge gap limits the ability of clinicians to optimize exercise programs for improving cardiorespiratory fitness in the post-treatment phase. Therefore, the primary aim of this study was to directly compare the effects of a 12-week G-AEx protocol versus a CL-AEx protocol on measures of cardiorespiratory fitness and functional performance. We hypothesized that the G-AEx protocol, by systematically applying the principle of progressive overload, would elicit superior improvements in cardiorespiratory fitness compared to the CL-AEx protocol over the 12-week intervention period.

## 2. Materials and Methods

### 2.1. Design/Ethics Overview

This was a randomized controlled single-blind trial, with a prospective design, that took place at the Cardiorespiratory Assessment Unit and Physical Rehabilitation Center of Prince Sattam bin Abdulaziz University (PSAU), Al-Kharj, KSA, spanning from December 2023 to October 2024. Ethical approval for this trial (Protocol # RHPT/0023/0029) was granted by the PSAU’s Physical Therapy Research Ethics Committee on 26 November 2023. The study maintained compliance with the ethical standards established in the 1975 Declaration of Helsinki, incorporating all subsequent updates. Parents or legal representatives were provided with a thorough overview of the objectives, advantages, and possible risks of the study, and subsequently gave their informed consent before their children were enrolled. Participants also provided their assent. The trial was registered with ClinicalTrial.gov (ID: NCT07330141).

### 2.2. Study Power, Participants, and Randomization

#### 2.2.1. Power and Sample Size

The a priori sample size was determined using data from a preliminary study involving nine pediatric ALL survivors, where the standard deviation of peak VO_2_ change was observed to be 2.14 mL/kg/min, and the pooled within-group standard deviation was estimated at 4.59 mL/kg/min. For the initial planning phase, a conservative sample size calculation was performed using a one-way ANOVA paradigm in PASS software, version 23.0.2 (NCSS, Kaysville, UT, USA). This approach focused on detecting between-group differences at the post-intervention timepoint. To achieve a desired power of 90% with an alpha level of 0.05, this analysis indicated a need for 21 participants per group. Factoring in a projected 20% attrition rate, the recruitment target was set at 24 participants per group, for a total of 72 participants.

While the priori calculation was based on a one-way ANOVA, the primary analysis for this study employed a 3 (Group) × 2 (Time) mixed-model ANOVA to more appropriately test the group-by-time interaction effect. To ensure the study was sufficiently powered for this specific analytical model, a post hoc power analysis was conducted using the final sample size of 72. Based on the large observed effect size for the interaction on our primary outcome, peak VO_2_ (f = 0.47), this analysis confirmed that the achieved power was >0.98. This result demonstrates that the study was more than adequately powered to reliably detect the reported differences in the change among the G-AEx, CL-AEx, and control groups.

#### 2.2.2. Participants

A total of 72 pediatric survivors of ALL were enrolled from King Khalid Hospital (the hematology-oncology polyclinic), along with two additional referral facilities within the Riyadh region of KSA. The study population comprised children and adolescents between the ages of 10 and 18 who had completed their cancer treatments (including maintenance therapy). Cardiac structure and function were assessed via electrocardiography, with normal findings required for inclusion. Participants were further required to possess a motor strength grade of 3 or higher in the major muscle groups of lower extremities (i.e., the capacity to move through the complete testing range against gravity and maintain the position without added loads) [23], ensuring their physical readiness, no malformations of lower extremities or spine, and no history of regular exercise engagement within the preceding six months or plans to participate in any structured physical activity interventions for the duration of the study. A demonstrated willingness to participate in the exercise program was also necessary. Potential participants were deemed ineligible if they presented with secondary malignancies, considerable neurological or musculoskeletal disorders that could be reasonably expected to interfere with exercise program completion (e.g., severe neuropathy, impaired balance, balance problems, proprioceptive challenges, osteoporosis, or recurrent severe muscle cramps), or documented suboptimal cognitive performance (particularly in attention, executive functioning, and memory).

#### 2.2.3. Randomization and Group Assignment

An independent researcher, unaffiliated with this study, performed random allocation of the participating pediatric survivors of ALL into three intervention groups: G-AEx, CL-AEx, and control group (*n* for each group = 24). To ensure group equivalence and minimize bias, a permuted block randomization approach with varying block sizes was employed. Sequentially numbered, sealed opaque envelopes were prepared in each block. After the registration of each participant, the independent researcher advanced to open the next envelope in the designated block sequence to assign them to the respective intervention group.

### 2.3. Interventions

#### 2.3.1. Graded Aerobic Exercise

Participants in the G-AEx group engaged in a 12-week regimen, involving three sessions weekly, adhering to the procedural design referenced in previous research [8]. The program was administered under the guidance of an experienced exercise physiologist, who maintained strict compliance with the established exercise prescription standards [24]. The G-AEx protocol was implemented using a motorized treadmill (HP Cosmos Mercury^®^ Med, Nussdoerf-Traunstien, Germany), with adjustments made to suit each participant’s capabilities. During the initial two-week phase, sessions lasted 25 min, with the walking speed calibrated to 50% max HR. Subsequently, the intensity and duration were progressively increased by 5% max HR and 5 min at two-week intervals, ultimately reaching a target intensity of 75% max HR for 50 min by the final phase. The training intensity at the outset and its progressive adjustments were guided by max HR data collected during a pre-intervention ETT. Supplementary Table S1 provides a detailed breakdown of the G-AEx protocol and its progression.

In preparation for the G-AEx workout, participants undertook a 5 min warm-up consisting of a series of activities such as static and dynamic stretching, brisk treadmill walking, stationary cycling at no resistance, and controlled arm movement integrated with mindful breathing exercises. Also, a 5 min cool-down was integrated post-workout, featuring activities akin to the warm-up phase to help participants transition smoothly back to their pre-exercise condition. The HR was recorded at 5 s intervals throughout the training using a Polar S810i^TM^ monitor (Kempele, Finland). Participants were instructed to abstain from vigorous activities or food intake for at least two hours preceding the training. The decision to use the treadmill training in this study was based on its demonstrated advantages over alternative ergometers in previous studies, such as engaging a broad spectrum of muscles, ensuring full-body activity, and delivering a relatively comfortable experience during prolonged exercise.

#### 2.3.2. Constant-Load Aerobic Exercise

Participants assigned to the CL-AEx group participated in a moderate-intensity aerobic exercise regimen, calibrated to 65% of their max HR. Training sessions lasted 45 min and were administered three times weekly across a 12-week timeframe. All sessions were supervised by a qualified exercise therapist with over 10 years of professional experience. All sessions were performed on a treadmill (H/P/Cosmos Mercury Medical, H/P/Cosmos Sports & Medical GmbH, Nussdorf-Traunstein, Germany), providing a standardized and controlled setting. The intensity and duration of exercises were kept at a steady, uniform level throughout the program. Comprehensive preparation and safety measures, consistent with those applied to the G-AEx group, were implemented. These measures involved a standardized warm-up to ensure physical and mental readiness, real-time HR monitoring during the session, and a structured cool-down to facilitate post-exercise recovery.

#### 2.3.3. Standard Physical Rehabilitation

To ensure consistency across the study arms, participants in all three groups (G-AEx, CL-AEx, and Control) underwent an identical, supervised physical rehabilitation program. This standardized regimen was delivered by an oncology-certified physical therapist and was designed to optimize motor skills compromised by pediatric leukemia and its associated treatments. The program was conducted with a frequency of three sessions per week for a total duration of 12 consecutive weeks. Each session lasted approximately 45 min and was consistently supervised to ensure safety and adherence to the prescribed protocols. The program integrated the following components, which were structured according to the FITT-VP (Frequency, Intensity, Time, Type, Volume, and Progression) principles:

- Flexibility training: Static stretching focused on the gastro-soleus, hamstrings, hip flexors, and pectorals, complemented by active range-of-motion exercises for the upper/lower extremities and trunk. For static stretching, participants performed a 20 s hold followed by a 20 s release, repeated three times per muscle group. The active range of motion consisted of one set of 10 repetitions per joint/region. No specific progression in intensity or volume was implemented for flexibility training beyond consistent execution.
- Progressive resistance training: This training targeted major muscle groups (shoulders, pectorals, back, abdominals, gluteal muscles, and thighs) utilizing manual and mechanical resistance. The Volume was systematically increased to ensure progression. During the initial six weeks, participants performed two sets of eight repetitions per exercise. During the final six weeks, this was increased to four sets of 10 repetitions. All sets were separated by 2–3 min rest intervals.
- Balance training: A BOSU balance trainer (BOSU HEDSTROM Fitness, LLC, San Diego, CA, USA) was used by participants to perform a battery of exercises, including unilateral and bilateral stances, step-standing, squatting, and kneeling. The protocol also incorporated functional activities such as object tracking, multi-angle turning, stooping, and multi-directional lunging. Intensity was progressed by transitioning from “eyes open” to “eyes closed” conditions. For BOSU-based tasks, participants completed five repetitions with 10–30 s holds. Functional activities (lunging and turning) were performed for five repetitions per direction.
- Conditioning exercise: General conditioning was performed for 15 min per session using stationary cycling or slow-paced running. This component was maintained at a low-intensity (<50% of max HR) to serve as a standardized baseline for all participants prior to the group-specific aerobic treadmill protocols.

### 2.4. Outcome Measures

To ensure rigorous and unbiased assessment, all outcome measurements were conducted by a standardized protocol. An independent assessor, blinded to participant group allocation and not involved in the intervention delivery, performed all evaluations. This included the pre- and post-intervention measurements of the primary endpoint (cardiorespiratory fitness) and secondary endpoints (functional capabilities). Prior to baseline testing, participants attended an orientation session to ensure familiarity with all assessment procedures.

#### 2.4.1. Cardiorespiratory Fitness (Primary Endpoints)

The assessment of cardiorespiratory fitness was conducted via the McMaster incremental cycling protocol [25] on an electromagnetically controlled cycle ergometer (Excalibur Sport, Lode, The Netherlands). During this symptom-limited exercise tolerance test (ETT), breath-by-breath gas exchange and heart rate data were continuously collected to assess several key variables. The primary outcome, peak oxygen consumption (peak VO_2_), was defined as the highest 30 s average of oxygen consumption achieved during the test and expressed in mL/kg/min; an increase in this value represents improved cardiorespiratory fitness. Other variables included peak minute ventilation (VE), the highest volume of air expired per minute during the test, expressed in L/min. From these, we derived peak oxygen pulse (peak O_2_P), calculated as peak VO_2_ divided by maximum heart rate (mL O_2_/beat), where an increase indicates improved cardiac efficiency, and the ventilatory equivalent for oxygen (VE/VO_2_), the ratio of minute ventilation to oxygen consumption, which was measured to assess ventilatory efficiency, where a decrease signifies a favorable improvement. The respiratory exchange ratio (RER), calculated as the volume of carbon dioxide produced divided by the volume of oxygen consumed (VCO_2_/VO_2_), was used as a secondary criterion for maximal effort. Finally, we assessed maximum heart rate (max HR) in beats/minute and one-minute heart rate recovery (HRR_1_), defined as the absolute decrease in heart rate one minute after exercise cessation, where an increase reflects improved autonomic function. To ensure the validity and reliability of all ETTs, rigorous standardization protocols were implemented. Before each assessment, participants were provided with identical oral and written instructions detailing pre-test requirements. These included abstaining from large meals for at least three hours, refraining from caffeine intake for 24 h, and avoiding any strenuous physical activity on the day preceding the test. Participants were also advised to wear loose-fitting, comfortable clothing and appropriate athletic footwear. Furthermore, a comprehensive familiarization session was held prior to the baseline ETT to become acquainted with the testing methodology. To control for diurnal variation and other environmental factors, all pre- and post-intervention testing sessions for a given participant were scheduled at the same time of day and conducted under consistent laboratory conditions.

The test followed a ramp protocol, starting at 25 W. Every two minutes, the workload was elevated by either 12.5 W, 25 W, or 50 W, with the increment size determined based on individuals’ gender and height. The pedal rate was regulated to remain between 50 and 70 rpm. Upon completion of the test, a 3 min active cool-down was implemented, consisting of continued cycling at 25 W. Subsequently, participants experienced a 3 min passive recovery period. Verbal prompts were provided to encourage maximal exertion during the test, with the goal of sustaining the target cadence for as long as physiologically possible. A Med-graphics CPX/D system (St. Paul, MN, USA) portable gas analyzer was employed for continuous breath-by-breath analysis of expired gases. Simultaneous monitoring of cardiac activity was conducted using a 12-lead electrocardiogram (CASE^TM^ Exercise Testing, GE Healthcare Inc., Milwaukee, WI, USA). During the test, perceived exertion was measured via the 11-point children’s OMNI-cycle scale, which utilizes pictorial descriptors and a numerical scale from 0 (extremely easy) to 10 (extremely hard) [26]. The peak VO_2_ validation was based on the attainment of at least one of the following secondary criteria: a VO_2_ plateau (indicating no further increase in O_2_ uptake despite increased workload), observable signs of maximal exertion (e.g., facial flushing, hyperpnea, postural instability), a perceived exertion rating exceeding 7 on the OMNI-cycle scale, a RER of 1.10 or greater, or a max HR ≥ 85% of the age-predicted maximum HR [4,8,27].

#### 2.4.2. Functional Capabilities (Secondary Endpoints)

To characterize functional performance, three validated assessments were administered: the Six-Minute Walk Test (6MWT) to gauge walking capacity and endurance [28], the 4x10-Meter Shuttle Run Test (4x10-mSRT) to measure agility, movement speed, and coordination [29], and the Timed Up and Down Stairs (TUDS) test to evaluate functional mobility, which includes strength and endurance components [30]. To minimize learning effects that could influence baseline scores, a familiarization session was conducted prior to the formal pre-intervention assessment. During this session, participants performed one submaximal practice trial of each test to ensure they understood the instructions and were comfortable with the procedures. For all formal testing sessions, a 10–15 min rest period was provided between each assessment to mitigate the potential fatigue effects.

For the 6MWT, a 30 m straight and level corridor with clearly delineated reversal points was utilized. Participants were asked to walk at their normal walking speed for six minutes, aiming to maximize the distance traversed without running or jogging. Adhering to the American Thoracic Society, the researcher provided standardized encouragements (verbal cues) every minute [31], while also using a stopwatch to provide precise timing for the test. A measuring wheel (with the subsequent conversion from feet to meters) was used to measure the total distance walked, which served as the primary indicator of walking efficiency (i.e., the farther the distance walked, the higher the level of efficiency demonstrated in walking).

The 4x10 mSRT was performed using a 10-m track with clearly marked start and finish lines, consistent with a previous description [29]. Participants were instructed to run four round-trip loops along the track as rapidly as possible. At each line crossing, participants exchanged a sponge, either dropping one down or picking one up from a designated location behind the line. The timer, operated by a researcher positioned at the starting line, was stopped the moment the participant crossed the line with one foot. The recorded time, in seconds, represented the completion time for the task. Three trials were performed, with 1–2 min rest periods between trials, and the fastest trial was selected for the analysis.

The TUDS test involved a 14-step staircase with 20 cm high steps. Participants began with one foot off the lower landing and were asked to ascend and descend the stairs as quickly and safely as possible, turning around at the top. Participants were permitted to use any comfortable strategy for stair climbing, including run-ups, skipping steps, or alternating foot patterns. Verbal cues (ready, set, go) were used to initiate the task. The time to complete the task, in seconds, was measured using a stopwatch. Each measurement represents a single trial. Lower scores (faster times) reflect enhanced functional mobility [30].

### 2.5. Statistical Analysis

All statistical calculations were executed with Minitab 19.2 software (Minitab Inc., State College, PA, USA). A predefined alpha level of 0.05 was applied to determine statistical significance across all analyses. The assumption of normality of all data sets was evaluated using the Anderson–Darling normality test. To analyze the group-by-time interaction effect, a mixed model ANOVA with a 2 (time: pre/post) × 3 (group: G-AEx/CL-AEx/control) design was implemented. This analysis served to establish the significance of differences among the study groups, with time as a within-subjects factor and intervention group as a between-subjects factor. To delineate specific differences between group pairings, Tukey’s HSD test was implemented for post hoc analysis. In cases where ANOVA revealed a statistically significant interaction, paired exploratory *t*-tests were performed to quantify within-group variations across the two time points. To quantify the magnitude of statistically significant between-group differences, the partial eta-squared (η^2^P) statistic was employed. The effect size of significant changes observed within each group was evaluated through Hedge’s g formula.

## 3. Results

### 3.1. Enrollment and Retention

**Figure 1:**
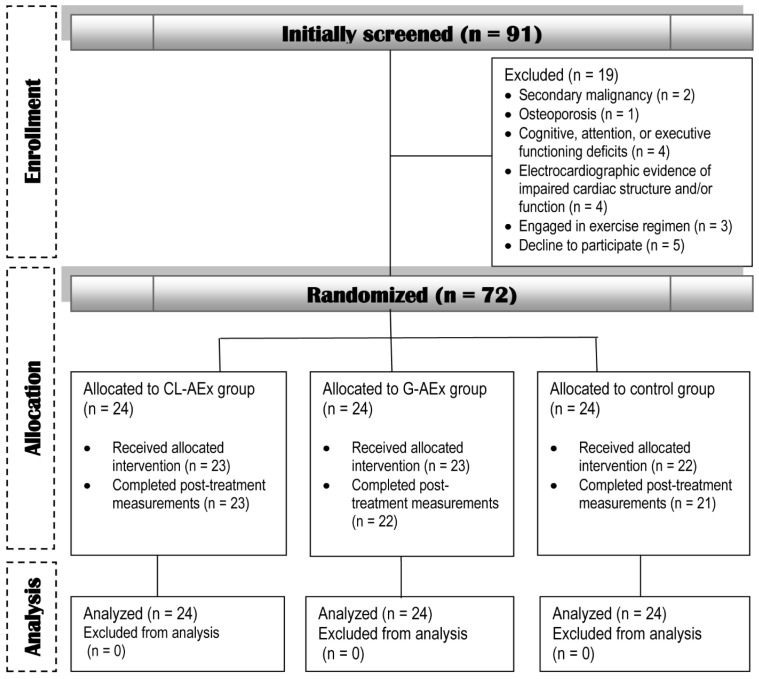
Flowchart illustrating the flow of study participants from initial recruitment through final data analysis according to the CONSORT guidelines.

The trajectory of study participants is illustrated in the CONSORT flow diagram (Figure 1). Among 92 survivors assessed for eligibility, 72 met the inclusion criteria and were randomized into three equal groups (*n* = 24 per group). Six participants (~8% of the total sample) were lost to follow-up and did not complete the post-intervention assessments. Specifically, attrition occurred in the G-AEx group (*n* = 2), the CL-AEx group (*n* = 1), and the control group (*n* = 3). Reasons for withdrawal included undisclosed personal considerations, scheduling conflicts, and travel outside the study region. In accordance with the intention-to-treat principle, missing post-intervention data were handled using multiple imputation by chained equations. Five imputed datasets were generated using a linear regression model incorporating baseline measures, group allocation, and demographic covariates (age and BMI). Results were subsequently pooled using Rubin’s Rules for the final analysis; thus, all 72 randomized participants were included in the final analytical model.

### 3.2. Compliance with Training

Compliance with the 12-week intervention was high across all three groups, with no significant difference observed in the percentage of sessions completed. Out of a total of 36 prescribed sessions, the median [interquartile range] compliance rate was 94.4% [91.7–97.4%] for the G-AEx group, 91.7% [88.9–94.4%] for the CL-AEx group, and 93.1% [88.9–94.4%] for the control group. A Kruskal–Wallis test confirmed that there was no statistically significant difference in the compliance rate among the three groups (H_2_ = 5.25, *p* = 0.07), indicating that compliance was comparable. Importantly, all groups demonstrated high fidelity to their assigned interventions, consistently completing the prescribed session durations and, for the G-AEx and CL-AEx groups, successfully meeting their target exercise intensities.

### 3.3. Baseline Comparability

The baseline assessments revealed that the G-AEx, CL-AEx, and control groups were well-matched concerning age, anthropometric indices, gender proportion, and pubertal maturity (*p* > 0.05 for all variables). Notably, the three groups also exhibited parity in key clinical variables related to ALL, including age of onset (*p* = 0.58), treatment protocols (*p* = 0.94), time elapsed since chemotherapy completion (*p* = 0.49), medication dosages [specifically, anthracycline (*p* = 0.96) and prednisone (*p* = 0.74)], and receipt of other therapies such as radiotherapy or hematopoietic stem cell transplantation (*p* = 0.77) (Table 1).

### 3.4. Differential Intervention Effects

The results of the 2 × 3 mixed-model ANOVA revealed significant moderate-to-large group-by-time interaction effects on the cardiorespiratory fitness markers peak VO_2_ (F_2,69_ = 31.01; *p* < 0.001; η^2^P = 0.47), VE (F_2,69_ = 14.59; *p* < 0.001; η^2^P = 0.29), VE/VO_2_ (F_2,69_ = 30.96; *p* < 0.001; η^2^P = 0.46), RER (F_2,69_ = 7.18; *p* = 0.002; η^2^P = 0.17), peak O_2_P (F_2,69_ = 19.42; *p* < 0.001; η^2^P = 0.36), Max HR (F_2,69_ = 6.19; *p* = 0.003; η^2^P = 0.15), and HRR_1_ (F_2,69_ = 4.27; *p* = 0.018; η^2^P = 0.11), indicating that the changes in these variables from pre- to post-intervention differed significantly across the study groups (Table 2). Post hoc pairwise comparisons using Tukey’s HSD test further clarified these differences, revealing that G-AEx group demonstrated significant improvements compared to both the CL-AEx group [peak VO_2_ (*p* = 0.002), VE (*p* = 0.015), VE/VO_2_ (*p* = 0.025), RER (*p* = 0.002), peak O_2_P (*p* = 0.03), max HR (*p* = 0.013), and HRR_1_ (*p* = 0.042)] and control group [peak VO_2_ (*p* = 0.021), VE (*p* = 0.002), VE/VO_2_ (*p* = 0.018), RER (*p* < 0.001), peak O_2_P (*p* = 0.013), max HR (*p* = 0.002), and HRR_1_ (*p* = 0.023)]. The CL-AEx group exhibited a greater magnitude of change compared to the control group; these differences, however, did not reach statistical significance (all *p* > 0.05) (Table 3).

The analysis also identified a significant large group-by-time interaction effect on the functional capability metrics 6MWT (F_2,69_ = 26.77; *p* < 0.001; η^2^P = 0.44), 4x10-mSRT (F_2,69_ = 15.94; *p* < 0.001; η^2^P = 0.31), and TUDS (F_2,69_ = 37.52; *p* < 0.001; η^2^P = 0.52), suggesting that the pattern of change in these metrics from pre- to post-intervention varied significantly across the three groups (Table 4). Follow-up pairwise comparisons elucidated the observed differences. Specifically, the G-AEx group experienced better performance in all functional assessments as compared to the CL-AEx group [6MWT (*p* = 0.039), 4x10-mSRT (*p* = 0.008), TUDS (*p* = 0.015)] and the control group. [6MWT (*p* = 0.029), 4x10-mSRT (*p* = 0.034), TUDS (*p* = 0.037)]. The CL-AEx group displayed a greater mean change than the control group; these differences, however, were not found to be statistically significant (all *p* > 0.05) (Table 5).

## 4. Discussion

This research was designed to assess the differential effects of CL-AEx and G-AEx on cardiorespiratory fitness and functional capacity among pediatric survivors of ALL. Among the key results, the G-AEx group experienced significant enhancements in all cardiorespiratory metrics (including peak VO_2_, VE, VE/VO_2_, RER, peak O_2_P, max HR, and HRR_1_), which were more pronounced than those in the CL-AEx and control groups. A further key observation from the data is the marked improvement in functional capacity within the G-AEx group, reflected by increased distance in the 6MWT distances and faster performance times in the 4x10mSRT and TUDS, surpassing the outcomes of the CL-AEx and control groups.

While the effectiveness of aerobic exercise for pediatric survivors of ALL has been widely studied [8,12,14,15,16], this study breaks new ground by undertaking a comparative analysis of a G-AEx regimen, tailored for intensity and duration, against a CL-AEx regimen. The focus is to determine which approach is more effective, engaging, and conducive to positive health outcomes, while better meeting the specific needs of pediatric survivors of ALL. Distinctly, the G-AEx regimen, structured around intensity and duration, followed a two-week cycle that started with exercise at 50% max HR for 25 min in the first two weeks and concluded with exercise at 75% max HR for 50 min in the final two weeks. The CL-AEx, on the other hand, entailed training at a stable intensity of 65% max HR for 45 min per session, consistently applied over a 12-week period. As such, the relevance of these results cannot be overlooked, as they deliver critical and previously unexplored evidence that can guide the optimization of aerobic training strategies for pediatric survivors of ALL, expediting progress in cardiorespiratory fitness and functional capabilities and contributing to a more comprehensive approach to rehabilitation and long-term care for this vulnerable population.

This study identified a notable result, indicating that G-AEx outperformed CL-AEx in boosting peak VO_2_ in pediatric survivors of ALL. Although the underlying mechanism is not definitely established, it is plausible to hypothesize that the observed enhancements in peak VO_2_ may stem from improved cardiac output and/or more efficient oxygen extraction. This hypothesis is reinforced by the elevated values of max HR and peak O_2_P, which provide robust evidence supporting this interpretation. It is also possible that the peak VO_2_ enhancement is associated with partial easing of the respiratory challenges these survivors faced. In our view, synthesizing both hypotheses offers a strong, coherent rationale. The G-AEx regimen may have prompted adaptive transformations within the respiratory system while also enhancing the muscular system’s capacity to extract and utilize oxygen more effectively during training [8,19,20,32,33]. The marked elevation in VE post-G-AEx is an additional noteworthy result. This effect may be explained by exercise-driven enhancements in respiratory rate, tidal volume, and breathing depth, all of which work in tandem to sustain physiological homeostasis [34,35]. A further significant result was the greater decline in the VE/VO_2_ ratio following G-AEx. This result points to a balanced interaction between perfusion and ventilation under this training regimen, characterized by a proportional rise in ventilation alongside improved oxygen utilization. In addition, the peak O_2_P, max HR, and HRR_1_ displayed a more substantial rise in response to G-AEx, signaling improved cardiovascular and hemodynamic function. These results provide further validation that cardiac limitations did not hinder the participation of this population in the training regimen, enabling them to engage in conditioning activities for longer durations.

The results appear to be in agreement with earlier research, underscoring the positive influence of G-AEx on cardiorespiratory capacity. In a recently undertaken clinical trial, researchers explored the effects of a progressive G-AEx program in a cohort of pediatric survivors of ALL. The regimen, similarly as implemented herein, began at 50% max HR for 25 min in the first two weeks and was systematically intensified over 12 weeks, culminating at 75% max HR for 50 min in the last two weeks. Remarkably, the trial documented significant improvements in all measured cardiorespiratory variables (specifically, peak VO_2_, VE, VE/VO_2_, RER, peak O_2_P, max HR, and HRR_1_) [8]. Moreover, the results of this study align with the existing evidence, highlighting a clear connection between G-AEx and enhanced cardiopulmonary capacity in children and adolescents with various health issues, such as those recovering from chest burn injuries or those who are obese and living with bronchial asthma [19,20,33].

In terms of functional capabilities, this study found that a 12-week G-AEx regimen markedly boosted the survivors’ performance in the 6MWT, 4x10mSRT, and TUDS. These results reinforce previous findings that showcased the functional and physical advantages of aerobic exercises in pediatric survivors of ALL [8,14,15,36]. The improvements observed in these functional tasks are intrinsically linked to the concurrent enhancements in cardiorespiratory fitness metrics, providing a clearer physiological underpinning for the functional gains. Specifically, the significant improvement in the 6MWT, a recognized measure of submaximal aerobic endurance and functional mobility, can be directly attributed to enhancements in central and peripheral cardiorespiratory capacity. A higher peak VO_2_, a primary indicator of aerobic fitness, signifies an increased ability of the cardiorespiratory system to deliver oxygen to working muscles and for muscles to extract and utilize that oxygen [37], directly translating to improved endurance during sustained activities like walking. Concurrent improvements in oxygen pulse, reflecting enhanced stroke volume and cardiac efficiency, further contribute to a greater oxygen transport capacity [38]. Additionally, improved ventilatory efficiency (e.g., lower VE/VO_2_) would reduce the respiratory workload, making sustained ambulation less fatiguing.

The enhanced performance in the 4x10mSRT, which demands rapid acceleration, deceleration, and changes in direction interspersed with short sprints, also reflects improved cardiorespiratory function. While agility and speed have prominent neuromuscular components, a robust aerobic base, characterized by a higher peak VO_2_ and faster HRR1, is crucial for repeated sprint ability and effective recovery between efforts [39,40]. Better cardiovascular recovery allows for quicker restoration of energy stores and removal of metabolic byproducts (e.g., lactate buffering capacity), delaying fatigue and improving overall performance in intermittent, high-intensity activities [41]. Similarly, gains in the TUDS, a composite measure of balance, agility, and mobility crucial for daily activities, benefit from an augmented cardiorespiratory reserve. Improved aerobic fitness reduces the physiological strain associated with transitions, turns, and quick movements, indirectly enhancing muscle power output and coordination during these tasks [8,42]. The synergistic interplay between a strengthened cardiorespiratory system and improved muscular function (as potentially indicated by increased peripheral muscle strength, though not directly measured here) allows for more efficient execution of daily functional activities, ultimately contributing to a more active and independent lifestyle. More conceivably, the interaction of these factors together could have been the driving force behind the observed functional outcomes, considering the multifaceted nature of the physiological adaptations elicited by aerobic exercise [8,19,20,33].

The statistically significant pre–post changes observed in certain outcomes within the control group, specifically improvements in RER, max HR, HRR1, and the 4x10m-SRT, warrant careful consideration. These gains, despite the absence of specific aerobic interventions, can be attributed to several factors. Firstly, learning and familiarization effects often manifest in functional performance tests such as the 4x10m-SRT, where repeated exposure to the task can lead to improved coordination, pacing, and efficiency, thereby enhancing performance independent of true physiological adaptation [43]. Similar learning effects, though less pronounced, can influence self-paced or effort-dependent components of cardiorespiratory assessments, potentially allowing participants to optimize their performance strategy during the post-intervention ETT. Secondly, variability in maximal cardiopulmonary exercise testing effort across time points could contribute to these observations. While participants were encouraged to exert maximal effort during both pre- and post-intervention ETTs, the intrinsic variability in motivation, perceived exertion, or even transient daily physiological states (e.g., fatigue, sleep quality) can subtly influence outcomes like peak HR and RER, which are direct indicators of effort and metabolic strain [44,45]. Apparent improvements could reflect instances where post-intervention testing captured a genuinely higher effort level than baseline, rather than a physiological training effect. Thirdly, despite instructing control participants to maintain their usual habitual physical activity, unmeasured changes in activity outside the intervention cannot be entirely ruled out. Although participants affirmed no participation in new structured programs, subtle increases in spontaneous physical activity, engagement in recreational pursuits, or general lifestyle modifications over the 12-week period could have contributed to minor improvements in cardiorespiratory fitness and functional capacity [42]. Such low-level, unquantified physical activity, combined with developmental maturation often ongoing in this pediatric population, may collectively lead to detectable, albeit modest, physiological and performance improvements [46]. Finally, the observed improvements may represent a true, albeit small, physiological adaptation resulting from the active control intervention. Despite not being aerobically focused, the structured, multi-component nature of the intervention provided a physiological stimulus that could plausibly account for observed improvements in select parameters of cardiorespiratory fitness in this deconditioned population.

### 4.1. Strengths and Limitations

This study is underpinned by a rigorous methodological approach, featuring a randomized controlled single-blind design. The sample size was determined in advance to achieve a balance between statistical power and practical constraints, with a resulting power of 90%. These methodological advantages collectively amplify the study’s ability to infer causality, yield dependable findings, and offer meaningful contributions to the field of inquiry. Moreover, the treatment priorities and data compiled—particularly regarding cardiorespiratory capacity and functional performance metrics—reflect the central clinical consideration of ALL survivors, their families, and clinicians, whether in the short- or long-term aftermath of the medical treatment. In addition, compliance with the treatment protocols was notably high across the three groups, and there were only minimal reports of any adverse outcomes related to the delivered training modules.

While this study advances understanding of the research question, it is imperative to address its limitations to ensure comprehensive and context-sensitive interpretation and exploration of the results. First, regarding generalizability, the findings are based on a relatively small sample size, which may limit statistical power for secondary outcomes and temper the generalizability of the results. However, it is important to note that participants were recruited from three different medical centers, which enhances the representativeness of our sample and strengthens the external validity compared to a single-center study. Nevertheless, our study exclusively included survivors aged 10–18 years who had completed all medical treatments, meaning the results may not extend to younger survivors or those still undergoing therapy. Second, several methodological limitations should be noted. We did not employ objective measures, such as accelerometry, to monitor participants’ physical activity levels outside of the supervised sessions. Therefore, we cannot completely rule out the possibility that unmeasured changes in habitual activity may have influenced the outcomes. The study also did not incorporate psychosocial measures to assess factors like participant motivation, enjoyment, or perceived barriers related to each exercise protocol. The inclusion of such measures in future research would provide valuable context regarding the feasibility and potential for long-term adherence. Third, while randomization was successful, a significant proportion (41.7%) of the G-AEx group had undergone radiotherapy or hematopoietic stem cell implantation. Although this did not appear to blunt their training response, the potential for treatment history to act as an effect modifier warrants consideration. Finally, as this study did not include a long-term follow-up, it does not provide verification of the sustained effects of the interventions. Investigating the long-term implications of these training protocols remains a critical focus for future research.

### 4.2. Clinical and Research Implications

The results presented in this study hold significant implications for bridging the gap between research and clinical practice, offering valuable insights for designing effective interventions for pediatric survivors of ALL. This research provides a foundation for evidence-based guidelines to create personalized exercise interventions that can effectively enhance rehabilitation outcomes in this population. The results imply that a structured, progressive aerobic training approach may be the optimal strategy for enhancing cardiorespiratory and functional capacity in this patient group. The G-AEx outperformed CL-AEx in improving key aerobic fitness indicators. This highlights the advantages of progressively increasing exercise intensity and duration, which facilitates better patient adaptation to aerobic exercises. Such a progressive approach likely supports sustained physiological adaptations in the cardiorespiratory system, ultimately improving exercise tolerance and functional capacity. These results suggest that clinicians and rehabilitation experts working with pediatric survivors of ALL should consider incorporating G-AEx into a comprehensive treatment plan, given its superior effectiveness over CL-AEx. The study calls for further research to uncover the physiological mechanisms underlying the enhanced effectiveness of the G-AEx over CL-AEx in ALL survivors. Future research could also explore the influence of G-AEx on broader health indicators, such as metabolic profiles and inflammatory biomarkers, to gain a more holistic perspective on its therapeutic potential, enabling the design of tailored rehabilitation strategies that cater to the diverse needs of this population.

## 5. Conclusions

This study provides preliminary evidence regarding short-term exercise strategies for pediatric survivors of ALL. Our comparative analysis indicates that a 12-week G-AEx protocol may result in greater improvements in cardiorespiratory fitness and functional capacity compared to a CL-AEx approach within the immediate post-intervention period. These findings offer an initial evidence-based framework for refining aerobic exercise prescriptions in this specific clinical population. However, given the absence of long-term follow-up, future longitudinal research is necessary to determine the durability of these improvements and to further explore the underlying physiological mechanisms. This work supports the ongoing development of tailored, evidence-informed rehabilitation programs aimed at enhancing the functional recovery of pediatric ALL survivors during the survivorship phase.

## Figures and Tables

**Table 1:** Baseline demographic and clinical characteristics of the study participants.

Variables	G-AEx Group (*n* = 24)	CL-AEx Group (*n* = 24)	Control Group (*n* = 24)	*p*-Value
Age, year	14.38 ± 2.20	14.25 ± 1.85	15.13 ± 2.17	0.29 ^a^
Sex (M/F), *n* (%)	17 (70.8)/7 (29.2)	15 (62.5)/9 (37.5)	13 (54.2)/11 (45.8)	0.54 ^b^
Weight, Kg	49.33 ± 10.51	48.33 ± 8.42	49.79 ± 8.78	0.86 ^a^
Height, m	1.46 ± 0.13	1.44 ± 0.11	1.48 ± 0.12	0.45 ^a^
BMI, Kg/m^2^	22.71 ± 1.29	23.11 ± 1.12	22.44 ± 1.26	0.17 ^a^
Age of onset, years	9.12 ± 2.13	8.79 ± 1.88	9.37 ± 1.74	0.58 ^a^
Post-treatment time, month	29.30 ± 7.54	30.46 ± 6.57	31.83 ± 7.77	0.49 ^a^
Treatment protocol, *n* (%)				
UKALL	4 (16.7)	3 (12.5)	5 (20.8)	0.94 ^b^
CCG-1991	4 (16.7)	2 (8.3)	3 (12.5)	
CCG-1961	2 (8.3)	4 (16.7)	3 (12.5)	
CCG-1891	5 (20.8)	4 (16.7)	6 (25)	
CCG-1882	9 (37.5)	11 (45.8)	7 (29.2)	
Medication dosages				
Anthracycline, mg/m^2^	133.13 ± 74.45	131.25 ± 36.52	136.87 ± 61.49	0.96 ^a^
Prednisone, mg/m^2^	5121.67 ± 3074.44	4503.33 ± 3325.24	5180.00 ± 3365.65	0.74 ^a^
Other therapies (RT/HSCT), *n* (%)	7 (29.2)/3 (12.5)	5 (20.8)/4 (16.7)	9 (37.5)/2 (8.3)	0.77 ^b^

Continuous variables are listed as mean ± StDev while categorical variables are presented as frequency (%). G-AEx: graded aerobic exercise, CL-AEx: constant-load aerobic exercise, BMI: body mass index, M/F: male/female, RT: radiotherapy, HSCT: hematopoietic stem cell transplantation, CCG: Children’s Cancer Group protocols, UKALL: United Kingdom Acute Lymphoblastic Leukemia protocols. ^a^ One-way ANOVA test, ^b^ Fishers’ exact test.

**Table 2:** Changes in cardiorespiratory fitness variables across the study groups.

	G-AEx Group (*n* = 24)	CL-AEx Group (*n* = 24)	Control Group (*n* = 24)	G-by-T Interaction	
				*p*-Value	η^2^P
Peak VO_2_, mL/kg/min					
Pre	28.83 ± 2.27	29.13 ± 3.66	31.02 ± 2.80	<0.001 *	0.47
Post	36.91 ± 3.06	32.25 ± 2.16	31.47 ± 3.33		
*p*-value	<0.001 *	0.0008 *	0.31		
Hedges’ g (95% CI)	2.89 (1.96–4.02)	0.99 (0.42–1.64)	0.14 (0.13–0.43)		
VE, L/min					
Pre	74.53 ± 5.26	75.44 ± 3.25	74.36 ± 3.76	<0.001 *	0.29
Post	83.34 ± 5.89	76.61 ± 4.82	75.92 ± 5.85		
*p*-value	<0.001 *	0.14	0.12		
Hedges’ g (95% CI)	1.52 (0.87–2.26)	0.27 (0.09–0.65)	0.31 (0.08–0.71)		
VE/VO_2_					
Pre	43.10 ± 3.50	42.04 ± 4.77	41.33 ± 4.01	<0.001 *	0.46
Post	33.42 ± 4.20	39.70 ± 5.61	40.71 ± 4.60		
*p*-value	<0.001 *	0.012 *	0.29		
Hedges’ g (95% CI)	2.24 (1.59–3.40)	0.43 (0.10–0.79)	0.14 (0.12–0.41)		
RER					
Pre	1.10 ± 0.05	1.12 ± 0.06	1.11 ± 0.04	0.002 *	0.17
Post	0.96 ± 0.09	1.03 ± 0.08	1.06 ± 0.06		
*p*-value	<0.001 *	<0.001 *	0.0002 *		
Hedges’ g (95% CI)	1.86 (1.13–2.69)	1.23 (0.61–1.92)	0.95 (0.49–1.45)		
Peak O_2_P, mL O_2_/beat					
Pre	7.51 ± 1.30	7.52 ± 1.17	7.47 ± 1.21	<0.001 *	0.36
Post	9.18 ± 1.34	7.80 ± 1.09	7.64 ± 0.86		
*p*-value	<0.001 *	0.036 *	0.28		
Hedges’ g (95% CI)	1.22 (0.72–1.78)	0.24 (0.02–0.47)	0.16 (0.14–0.46)		
Max HR, beat/min					
Pre	183 ± 8	181 ± 7	182 ± 7	0.003 *	0.15
Post	197 ± 7	190 ± 8	187 ± 8		
*p*-value	<0.001 *	<0.001 *	0.0003 *		
Hedges’ g (95% CI)	1.80 (1.19–2.51)	1.16 (0.57–1.81)	0.64 (0.27–1.05)		
HRR_1_, beat/min					
Pre	32 ± 7	29 ± 8	30 ± 7	0.018 *	0.11
Post	39 ± 6	34 ± 7	32 ± 6		
*p*-value	<0.001 *	0.001 *	0.0003 *		
Hedges’ g (95% CI)	1.04 (0.63–1.50)	0.64 (0.29–1.03)	0.30 (0.07–0.54)		

Data are displayed as (mean ± StDev). Abbreviations: G-AEx: graded aerobic exercise, CL-AEx: constant-load aerobic exercise, G-by-T: group-by-time, CI: confidence interval, VO_2_: oxygen uptake, VE: peak minute ventilation, VE/VO_2_: ratio of minute ventilation to oxygen consumption, RER: respiratory exchange ratio, O_2_P: oxygen pulse, Max HR: maximum heart rate maximum, HRR_1_: one-minute heart rate recovery. η^2^P: effect size for the between-group difference, Hedges’ g: effect size for the within-group difference, * significant at *p* ˂ 0.05.

**Table 3:** Pairwise comparisons of estimated marginal means for cardiorespiratory fitness outcomes by group.

Variable	G-AEx vs. Control		CL-AEx vs. Control		G-AEx vs. CL-AEx	
	MD	*p*-Value	MD	*p*-Value	MD	*p*-Value
Peak VO_2_, mL/kg/min	1.63	0.021 *	–0.55	0.42	2.18	0.002 *
VE, L/min	3.90	0.002 *	0.89	0.45	2.91	0.015 *
VE/VO_2_	−2.77	0.018 *	−0.15	0.89	−2.63	0.025 *
RER	−0.05	<0.001 *	−0.01	0.58	−0.04	0.002 *
Peak O_2_P, mL O_2_/beat	0.79	0.013 *	0.10	0.74	0.69	0.03 *
Max HR, beat/min	6	0.002 *	2	0.45	5	0.013 *
HRR_1_, beat/min	4	0.023 *	1	0.79	4	0.042 *

Abbreviations: G-AEx: graded aerobic exercise, CL-AEx: constant-load aerobic exercise, VO_2_: oxygen uptake, VE: peak minute ventilation, VE/VO_2_: ratio of minute ventilation to oxygen consumption, RER: respiratory exchange ratio, O_2_P: oxygen pulse, Max HR: maximum heart rate maximum, HRR_1_: one-minute heart rate recovery. Note: Estimated Marginal Means are the group means averaged across both the pre- and post-intervention time points. * Significant at ***p*** < 0.05.

**Table 4:** Changes in functional capability metrics across the study groups.

	G-AEx Group (*n* = 24)	CL-AEx Group (*n* = 24)	Control Group (*n* = 24)	G-by-T Interaction	
				*p*-Value	η^2^P
6MWT, m					
Pre	452.17 ± 67.82	447.04 ± 64.81	450.33 ± 62.83	<0.001 *	0.44
Post	537.63 ± 76.10	466.54 ± 61.17	457.75 ± 60.01		
*p*-value	<0.001 *	0.02 *	0.24		
Hedges’ g (95% CI)	1.15 (0.74–1.62)	0.29 (0.05–0.56)	0.12 (0.08–0.32)		
4x10-mSRT, second					
Pre	16.40 ± 1.67	16.77 ± 1.30	15.92 ± 1.61	<0.001 *	0.31
Post	12.98 ± 1.16	14.53 ± 1.52	14.97 ± 1.28		
*p*-value	<0.001 *	<0.001 *	0.0003 *		
Hedges’ g (95% CI)	2.30 (1.56–3.18)	1.53 (0.88–2.26)	0.63 (0.27–0.99)		
TUDS, second					
Pre	15.48 ± 2.33	14.97 ± 1.56	14.59 ± 1.67	<0.001 *	0.52
Post	11.44 ± 1.73	14.14 ± 1.76	14.20 ± 1.25		
*p*-value	<0.001 *	0.027 *	0.11		
Hedges’ g (95% CI)	1.90 (1.29–2.63)	0.48 (0.03–0.95)	0.25 (0.06–0.57)		

Data are summarized as (mean ± StDev). Abbreviations: G-AEx: graded aerobic exercise, CL-AEx: constant-load aerobic exercise, G-by-T: group-by-time, CI: confidence interval, 6MWT: Six-Minute Walk Test, 4x10-mSRT: 4x10-Meter Shuttle Run Test, TUDS: Timed Up and Down Stairs. η^2^P: effect size for the between-group difference, Hedges’ g: effect size for the within-group difference, * significant at *p* ˂ 0.05.

**Table 5:** Pairwise comparisons of estimated marginal means for functional capability outcomes by group.

Variable	G-AEx vs. Control		CL-AEx vs. Control		G-AEx vs. CL-AEx	
	MD	*p*-Value	MD	*p*-Value	MD	*p*-Value
6MWT, m	40.83	0.029 *	2.75	0.88	38.10	0.039 *
4x10-mSRT, second	−0.76	0.034 *	0.20	0.56	−0.96	0.008 *
TUDS, second	−0.93	0.037 *	0.17	0.44	−1.10	0.015 *

Abbreviation: G-AEx: graded aerobic exercise, CL-AEx: constant-load aerobic exercise, MD: mean difference, 6MWT: Six-Minute Walk Test, 4x10-mSRT: 4x10-Meter Shuttle Run Test, TUDS: Timed Up and Down Stairs. Note: Estimated Marginal Means are the group means averaged across both the pre- and post-intervention time points. * Significant at *p* < 0.05.

## Data Availability

The data presented in this study are available on request from the corresponding author due to privacy and ethical restrictions associated with the informed consent process for this vulnerable pediatric population.

## Supplementary Materials

The following supporting information can be downloaded at: https://www.mdpi.com/article/10.3390/healthcare14050608/s1, Table S1: A detailed breakdown of the G-AEx protocol and its progression.

## Abbreviations

The following abbreviations are used in this manuscript:

4x10-mSRT	4x10-Meter Shuttle Run Test
6MWT	Six-Minute Walk Test
ALL	Acute lymphoblastic Leukemia
ANOVA	Analysis of Variance
CL-AEx	Constant-Load Aerobic Exercise
ETT	Exercise Tolerance Test
G-AEx	Graded Aerobic Exercise
HR	Heart Rate
HRR_1_	One-Minute Heart Rate Recovery
HSD	Honest Significant Difference
O_2_P	Oxygen Pulse
RER	Respiratory Exchange Ratio
SD	Standard Deviation
TUDS	Timed Up and Down Stairs
VE	Peak Minute Ventilation
VE/VO_2_	Ratio of Minute Ventilation to Oxygen Consumption
VO_2_	Oxygen Consumption
