# Supplementary material for: Graded Versus Constant-Load Aerobic Exercise in Pediatric Leukemia Survivors: A 12-Week RCT on Cardiorespiratory Fitness and Functional Performance
Source: Healthcare (Basel). 2026 Feb 27;14(5):608. doi: 10.3390/healthcare14050608 (PMC12985029; doi:10.3390/healthcare14050608)
Supplement: Supplementary file 1 [file healthcare-14-00608-s001.zip › healthcare-4091843-supplementary.pdf]

Supplementary Table S1: A detailed breakdown of the G-AEx protocol and its progression

Week No.	G-AE intensity (% of HR_{max})	G-AE duration (*min*)	Entire session time (*min*)	Rest intervals (*n*)
Wks. 1 – 2	50 %	25	35	2
Wks. 3 – 4	55 %	30	40	3
Wks. 5 – 6	60 %	35	45	4
Wks. 7 – 8	65 %	40	50	4
Wks. 9 – 10	70 %	45	55	5
Wks. 11 – 12	75 %	50	60	5

G-AE: graded aerobic exercise; Wks.: weeks; min: minutes, *n*: number, HR_{max}: heart rate maximum,
Note: Training intensities were calculated based on the HR_{max} observed during the pre-treatment exercise tolerance test.

Methodological Notes:

Protocol Monitoring: All sessions were supervised one-on-one by a physical therapist. Target exercise intensity was continuously monitored using a chest-strap heart rate monitor (Polar H10, Polar Electro, Finland). The therapist's goal was to maintain the participant's heart rate within ±5 beats/min of the prescribed target for each interval.

Intensity Adjustments: If a participant's heart rate deviated from the target zone, the therapist made real-time adjustments to the treadmill speed. If the heart rate was too high, intensity was slightly decreased; if too low, intensity was incrementally increased.

Rest Intervals: The "rest intervals" listed in the table refer to the number of brief, active recovery periods; 1-2 minutes each. During these intervals, participants walked at a low intensity until their heart rate returned to the lower bound of the target heart rate zone, at which point the next work interval would commence. This did not represent a full stop or passive rest.
